# Relational Coordination and Organisational Social Capital Association with Characteristics of General Practice

**DOI:** 10.1155/2014/618435

**Published:** 2014-06-19

**Authors:** Sanne Lykke Lundstrøm, Kasper Edwards, Thomas Bøllingtoft Knudsen, Pia Veldt Larsen, Susanne Reventlow, Jens Søndergaard

**Affiliations:** ^1^Technical University of Denmark, DTU Management Engineering, Produktionstorvet, Building 424, 2800 Kongens Lyngby, Denmark; ^2^Research Unit of General Practice, University of Southern Denmark, J.B. Winsløws Vej 9, 5000 Odense C, Denmark; ^3^Research Unit for General Practice, University of Copenhagen, Center for Sundhed og Samfund, Øster Farimagsgade 5, Postboks 2099, 1014 København K, Denmark

## Abstract

*Background.* Relational coordination (RC) and organisational social capital (OSC) are measures of novel aspects of an organisation's performance, which have not previously been analysed together, in general practice. *Objectives.* The aim of this study was to analyse the associations between RC and OSC, and characteristics of general practice. *Methods.* Questionnaire survey study comprising 2074 practices in Denmark. *Results.* General practitioners (GPs) rated both RC and OSC in their general practice higher than their secretaries and nurses. The practice form was statistically significantly associated with high RC and OSC. RC was positively associated with the number of patients listed with a practice per staff, where staff is defined as all members of a practice including both owners and employees. *Conclusion.* The study showed that RC and OSC were significantly associated with type of profession and practice type. RC was also found to be significantly positively associated with number of patients per staff. However, the low response rate must be taken into consideration when interpreting the self-reported results of this study.

## 1. Introduction

General practice provides cost-efficient, first-line service and mindful of gatekeeping [1]. Still, studies have shown substantial variation of practice patterns in, for example, use of spirometry testing, prescribing of narrow-spectrum penicillin, and management of hypertension and number of different drugs prescribed per practice [2, 3]. These variations have only for a small part been explained by practice or physician characteristics like GP's gender and age, practice list size, structure, and workload [4]. Until now, focus has been on the above-mentioned easily measurable characteristics of general practice and the way they contribute to our understanding of differences in practice patterns. However, such characteristics may only to a minor extent serve as proxies for more subtle features. While relational coordination (RC) and organisational social capital (OSC) have not previously been jointly analysed in general practice; they have been shown to be related to an organisation's performance and have individually received much attention in health care and private industry with potential managerial implications.

Five key components of Danish general practice are as follows [1]:

1. list system with an average of 1600 persons per GP,
2. first-line provider and gatekeeper,
3. weekend and out-of-hours service,
4. 75% fee for service,
5. private, but publicly funded. Controlled through biannual contracts and negotiations between GP organisation and region.

Consider the following Facts:

- population of Denmark 5.5 million,
- five Regions,
- 2074 general practices.

RC was first studied in the airline industry and later within health care [5, 6]. RC is a tool for measuring and analysing the communication and relationship networks through which work is coordinated across functional and organisational boundaries [7]. In hospital settings, a positive association between RC and quality of care has been found [5]. Studies in primary care have emphasised the importance of enhancing RC between healthcare professionals and the fact that it may improve delivery of medical services [8, 9]. RC is defined as a mutually reinforcing process of interactions between communication and relationships carried out for the purpose of task integration. Studies have shown that RC is correlated with on-time airport departures and surgical performance [5–7], which have led to RC being perceived as a means of improving quality and performance under conditions of task interdependence, uncertainty, and time constraints [7, 10]. RC proposes that three relational dimensions contribute to effective coordination: shared goals, shared knowledge, and mutual respect [5]. These relational dimensions are theorised to enhance communication, that is, frequent, timely, accurate, and problem-solving, rather than blaming, thus making an organisation that can coordinate collective action [5, 11].

OSC is used when analysing the psychosocial work environment in organisations. OSC is closely related to social relations and networks [9] and is seen as a powerful resource for improving organisational performance [10]. OSC is defined as the ability for members in an organisation to collaborate, when solving the key task of the organisation [11]. OSC can also facilitate changes in the levels of trust between employees and owners and enhance cooperation and feelings of justice [11]. People in trusting relationships seek input from one another and they allow others to do their job without unnecessary supervision [12]. Having high OSC can therefore make it easier for different professions to collaborate and achieve a high level of RC. The work of a general practice is quite different from the airline and production industry where RC and OSC have their origin; still we believe that RC and OSC may offer new insight and opportunity for general practice to learn.

To improve RC and OSC in general practice, a deeper understanding of some main features of the general practice contribution to RC and OSC is needed. Practice structure such as single-handed, partnership, and cooperative practices is also associated with quality of care delivered, as is the workload. However, still no one has explored relationships between RC and OSC and how these measures are associated with general practice characteristics. Hence, this paper aims to (1) determine association between RC and OSC and (2) to explore associations between practice characteristics and RC and OSC, respectively.

## 2. Methods

### 2.1. Study Design

A questionnaire survey was carried out among 2074 Danish general practices from June to September 2011. The Organisation of General Practitioners provided addresses for all 2074 Danish practices. Danish registers contain information on the number of GPs in each practice, but no records are kept about other types of healthcare professions.

The questionnaire was designed to measure the psychosocial work environment and the task-based relationship ties in general practice. It comprised questions from two validated questionnaires: the RC survey [7] and the Copenhagen Psychosocial Questionnaire (COPSOQ) [13]. The questions from the RC Survey were translated from English into Danish through a crosscultural adaption process [14]. Firstly it was forward-translated by the first author and discussed within a multidisciplinary research group. Secondly, a professional translator subsequently made a back-translation. Thirdly, Jody Hoffer Gittell, the developer of the RC Survey, then evaluated the back-translated survey with emphasis on conceptual and cultural equivalence, rather than on linguistic equivalence. All questions were answered on a 5-point Likert scale.

The questionnaire was pilot tested in the autumn of 2010 and the spring of 2011 in two Danish general practices. Participants completed the questionnaire and were asked to comment on content, wording, and intelligibility. Only minor changes were made. The questions included in the present study will be described in detail later.

A letter including questionnaires and a stamped reply envelope was sent to the secretary in each general practice in Denmark. The practice secretary was asked to distribute the questionnaires among the owner(s) and the employee(s), fill in a background form with information about the practice, collect, and return all questionnaires and the background form. Nonrespondents received two reminders, the second one with new questionnaires, background form, and a stamped reply envelope.

### 2.2. Measures

In the RC Survey seven questions (1.1–1.7) measured the following dimensions of RC: frequent, timely, and accurate communication; the problem-solving nature of communication; and the degree to which relationships were characterised by shared goals, shared knowledge, and mutual respect [15]. Respondents were asked to answer each of the questions with respect to each of the other professions (GP, nurse, and secretary) within a general practice with respect to patients with chronic diseases; see Table 1. Caring for patients with chronic diseases in Danish general practice is usually organised around the secretary, who is the first point contact and relays the relevant information to the GP and/or other health personnel.

RC was calculated as a mean of the seven dimensions.

OSC was measured by means of statements about trust, justice, and cooperation. The trust scale comprises five statements (items 2.1–2.5) selected from the dimensions of “trust regarding management” and “mutual trust between employees” in COPSOQ II [13]. This scale has been validated on a representative sample of 3517 Danish employees [13]. The five statements are shown in Table 2. The justice scale comprises three statements. Items 3.1 and 3.2 were selected from the dimension “justice” in COPSOQ II [13]. For item 3.3 a negation of the original question from COPSOQ [16] was used in order to check consistency and make the respondents use both extremes of the 5-point Likert scale; see Table 2. The cooperation scale comprises three ad hoc statements, which were tested in the pilot study. Items 4.1–4.3 from Table 2 were used to assess the cooperation between employees.

### 2.3. Statistical Analysis

Two types of analyses were conducted: one where RC and OSC, respectively, were based on individual ratings and a second where they were based on practice average ratings. The analyses on individual ratings were adjusted for practice cluster effects using robust cluster estimation.

To analyse associations between RC and OSC, respectively, and a number of personal and organisational explanatory variables, mean differences with 95% confidence intervals (CIs) were calculated by use of analysis of variance. As explanatory variables geographical location, gender, practice types (single-handed, cooperative, and partnership practice), profession, number of healthcare professionals at the practice, length of employment in general practice, gender of the respondent, and size of list population were considered. All explanatory variables were categorical variables. To account for possible confounding, fully adjusted analyses as well as univariate analyses were conducted. A residual analysis was performed to assess the model assumptions.

The percentage of missing values and nonrelevant answers was calculated for both RC and OSC. Furthermore, two sensitivity analyses were performed in the calculations of RC: (1) Missing values and nonrelevant answers in the dimensions comprising the RC dimensions were substituted by the mean of the observed values for the dimension and (2) missing values and nonrelevant answers were substituted by 0.2 less than mean of the observed values of the dimension.

All analyses were performed using Stata Release 11.0 (StataCorp, College Station, TX, USA). A *P* value of <0.05 was considered statistically significant.

## 3. Results

**Figure 1:**
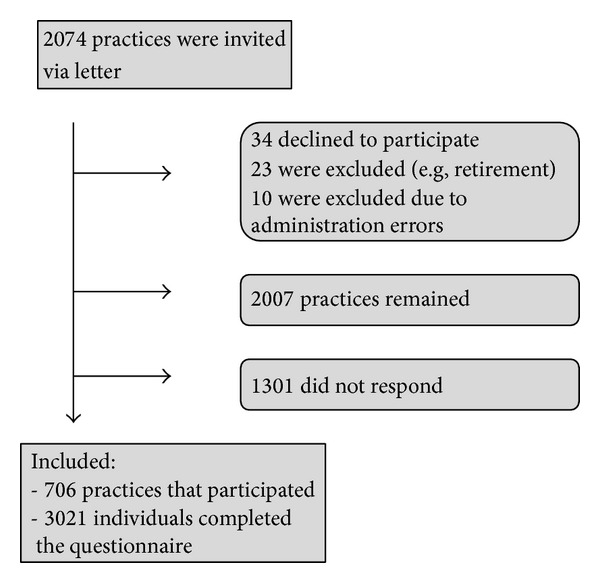
Flowchart.

Of the 2074 Danish general practices that were invited to participate, 706 (34%) general practices responded, Figure 1.

The study population is reported in Table 3.

The mean rating was 4.1 ± 0.3 (Mean ± SD) out of 5 and 80.3 ± 8.4 out of 100 for RC and OSC, respectively.

### 3.1. Personal Characteristics Associated with Ratings of RC and OSC

Table 4 shows a statistically significant association between profession and ratings of RC and OSC, respectively. GPs rated both RC and OSC higher than nurses and secretaries. GPs owning a general practice also rated RC higher than GPs who were employed (difference = −0.01; 95% CI −0.18 to 0.02).

Table 4 also shows a statistically significant association between RC and years of employment in general practice. Respondents who had been employed between 2 anf 5 years and between 6 and 10 years rated RC lower than respondents who had been employed less than 1 year in the same general practice, whereas respondents who had been employed more than 10 years rated RC higher than respondents with less than 1-year employment in the general practice.

Gender and age were not significant for the rating of RC or OSC.

### 3.2. Practice Characteristics Associated with Ratings of RC and OSC

Table 5 shows that practice form was highly statistically significantly associated with the ratings of both RC and OSC. Respondents from single-handed practices rated RC and OSC higher than respondents from other types of practices. Respondents from partnership practices had the lowest rating of RC and OSC.

The number of patients listed with a general practice per staff, where staff is defined as all members of a practice including both owner and employees, was statistically significant for the rating of RC in general practice. There was no difference in RC between practices with low and medium number of patients per staff (difference = 0.00; 95% CI −0.07 to 0.08). Practices with a high number of patients per staff rated RC higher than practices with a low number of patients per staff (difference = 0.14; 95% CI −0.04 to 0.24).

The number of patients listed with a general practice per GP was not statistically significant for ratings of RC or OSC, nor was the regional location of the practice.

### 3.3. Missing Values and Sensitivity Analysis

The percentage of missing values and nonrelevant answers for OSC statements was low, with a range of 0.43–5.71%. A higher frequency was seen for the RC questions, where the range was 6.15–18.12%. Both sensitivity analyses changed the effect of patients per staff ratio to nonsignificant.

## 4. Discussion

### 4.1. Main Findings

The results showed high OSC in Danish general practice (80.3 ± 8.4), when compared with the Danish national average of 64.9 [13]. There is no Danish national average for RC or any other benchmark to compare with. Instead, the RC measured in this paper (4.1 ± 0.3) is compared to the nine hospital studies presented in “High Performance Healthcare,” with RC ranging from 3.84 to 4.22 [5]. The average RC for Danish general practices presented in this paper is in the high end compared to the range from the hospital studies and is also with a smaller SD.

GPs rated both RC and OSC in their general practice higher than the secretaries and nurses. RC and OSC were both associated with practice types, where single-handed practices had higher ratings. Associations between profession and RC and OSC were also found. RC was also associated with the number of patients per staff in a general practice, a similar association was not found for OSC.

### 4.2. Interpretation

We believe that the higher ratings by the GPs may be due to the practices being owned and managed by GPs. GPs, in other words, have significant influence on both RC and OSC because they define processes and relationships.

There are mainly three types of general practices in Denmark: single-handed practice, cooperation practice, and partnership practice. Of the three types of practices single-handed practices had the highest ratings of RC and OSC compared to the other practice forms. Common for all practices is that they are owned and managed by GPs. Partnership and cooperative practices usually have more than one manager, and we hypothesise that such a joint leadership may be a source of confusion amongst the staff about who to report to. This may then cause uncertainty and lower levels of trust in general practice, resulting in the observed lower RC and OSC.

RC was found to increase when the number of patients per staff increased. Studies have shown that a high prevalence of polypharmacy (simultaneous use of five or more drugs) was found in practices characterised by a low patient load, probably meaning that the patients had high GP availability and employees had time for coordination and communication about everyday tasks [3]. High prevalence of polypharmacy could also be due to high level of contact between GPs and pharmaceutical delegates. However, access to data that could determine rapid contact between GPs and pharmaceutical delegates was not available. Ceteris paribus, we assume a relationship between numbers of patients per employee and time available per patient consultation; that is, with only few patients there are ample time for consultation. As the number of patients per staff increases there will be less time for consultation, discussion, and helping colleagues, which in turn should reduce RC. Nevertheless, this study shows that it is indeed possible to raise the number of patients per staff and also increase RC. Another important factor is the relationship between the patients and GP. However, the scope of this study was not to examine the effect of the patient-GP relationship. The findings indicate a point in the organisational development, where natural job specialisation will occur. The change comes from a place of need, more than from a growing focus on RC.

Geographical location, gender, and age were not associated with RC or OSC. It is remarkable that these factors, often hypothesised to be associated with performance such as quality of treatment and consultants per staff member in general practice, were not associated with RC or OSC. Instead, this paper shows that RC and OSC are associated with personal and practice characteristics.

### 4.3. Strengths and Limitations of the Study

Statements and questions used in this paper were from validated questionnaires [7–13, 17], which were tested in a pilot study. The discrepancy between our findings and the residual analyses indicated that the model assumptions were satisfied for both RC and OSC. The results of the sensitivity analysis suggest that RC with regard to the effect of patients per staff ratio should be further examined.

A limitation is that sample size calculations were not performed before sending out the survey. However, the large sample size with a total of 706 practices and 3021 individual respondents, the reasonably narrow confidence intervals, and the many statistically significant results, suggest that the sample size was sufficient for our study. Another limitation is the low response rate of 34%, which could lead to selection bias. As our paper considers associations rather than, for example, prevalence estimation, selection bias is unlikely to have affected our results significantly.

A general disadvantage of questionnaire-based surveys is the likelihood of social desirability response bias—people responding in a way that shows them in a good light. Particularly the owners of the general practices could be rating their practices well.

### 4.4. Implication for Future Research and Clinical Practice

More research is needed to achieve an in-depth exploration of the influence of RC and OSC on outcome performance measures, such as consultation rate per staff in each practice, characteristics of list populations, and patient satisfaction. Furthermore, it should be studied whether RC and OSC can be enhanced, both within general practice and between patients and healthcare professionals.

Even though increased RC in a general practice is hypothesised to reflect in communication with the patient and the service provided by the general practice future research should also include the patient. This is especially important due to the increasing focus on patient involvement in primary care.

## 5. Conclusion

This paper found a positive association between profession and RC and OSC in general practice. The paper also showed that single-handed practices have significantly higher RC and OSC than other practice types. Furthermore, the results showed a significantly positive association between RC and number of patients per staff.

## Figures and Tables

**Table 1:** Relational coordination questions.

Item	Dimension	Question
1.1	Frequent communication	How frequently do people in each of these groups communicate with you about patients with chronic diseases?
1.2	Timely communication	Do people in these groups communicate with you in a timely way about patients with chronic diseases?
1.3	Accurate communication	Do people in these groups communicate with you accurately about patients with chronic diseases?
1.4	Problem-solving communication	When problems occur with patients with chronic diseases, do the people in these groups blame others or work with you to solve the problem?
1.5	Shared goal	How much do people in these groups share your goals regarding patients with chronic diseases?
1.6	Shared knowledge	How much do people in each of these groups know about the work you do with patients with chronic diseases?
1.7	Mutual respect	How much do people in these groups respect the work you do with patients with chronic diseases?

**Table 2:** Organisational social capital statements.

Item	Scale	Statements
2.1	Trust	You can trust the information coming from the management
2.2	Trust	The management trusts that the employees do their work well
2.3	Trust	The employees do in general trust each other
2.4	Trust	Do employees withhold information from each other?
2.5	Trust	I am able to express my views and feelings to my colleagues
3.1	Justice	Conflicts between employees are resolved fairly for everybody involved
3.2	Justice	Work is distributed fairly
3.3	Justice	I do not have a large degree of influence over my work
4.1	Cooperation	Among us everybody is involved in decisions regarding changes
4.2	Cooperation	If I forget something, then one of my colleagues will take care of it for me
4.3	Cooperation	We have a good cooperation between workgroups

**Table 3:** Profile of the study population.

	Numbers of respondents
Gender	
Male	481
Female	1904
Professional position	
Secretary	674
Nurse	801
Physician-owner	1127
Physician-employed	253
Laboratory technologist	63
Others	75

**Table 4:** Associations of personal characteristics with individual ratings of relational coordination and organisational social capital.

	Relational coordination		Organisational social capital	
	Crude	Adjusted^A^	Crude	Adjusted^A^
	Difference	Difference [95% CI]	Difference	Difference [95% CI]
Years of employment in general practice	∗∗∗	∗		
Y < 1	—	—	—	—
2–5 Y	−0.5∗	−0.05 [−0.13; 0.02]	−2.02∗∗	−1.18 [−3.06; 0.71]
6–10 Y	−0.3	−0.05 [−0.14; 0.04]	−1.85∗	−2.31∗[−4.60; 0.28]
Y > 10	0.06∗	0.04 [−0.05; 0.13]	−0.75	−1.71 [−4.04; 0.61]
Profession	∗∗∗	∗∗∗		∗∗∗
GP owner	—	—	—	—
Secretary	−0.35∗∗∗	−0.37∗∗∗[−0.45; −0.29]	−4.15∗∗∗	−5.02∗∗∗[−6.96; −3.08]
Nurse	−0.11∗∗∗	−0.12∗∗∗[−0.18; −0.05]	−2.56∗∗∗	−3.94∗∗∗[−5.96; −1.93]
GP employed	−0.12∗∗∗	−0.1∗[−0.18; −0.02]	0.16	−0.97 [−3.46; 1.52]
Gender				
Male	—	—	—	—
Female	−0.14∗∗∗	−0.01 [−0.06; 0.04]	−2.62∗∗∗	0.56 [−1.04; 2.16]
Age				
Min–29 Y	—	—	—	—
30–39 Y	−0.16∗	−0.15∗[−0.27; −0.04]	−2.30	−1.35 [−4.56; 1.85]
40–49 Y	−0.10∗	−0.11 [−0.23; 0.02]	−2.94∗	−0.82 [−4.22; 2.58]
50–59 Y	−0.11∗	−0.15∗[−0.29; −0.02]	−2.71	−0.30 [−3.83; 3.23]
60–69 Y	−0.13∗	−0.16∗[−0.31; −0.01]	−2.26	−0.05 [−3.94; 3.84]
70–max	−0.09	−0.37 [−1.54; 0.81]	−1.58	5.40 [0.26; 11.06]

*P < 0.05,  **P < 0.01,  ***P < 0.001.

^A^A fully adjusted model including all variables listed in the table.

**Table 5:** Associations of practice characteristics with ratings for each general practice on relational coordination and organisational social capital, respectively.

	Relational coordination		Organisational social capital	
	Crude	Adjusted^A^	Crude	Adjusted^A^
	Difference	Difference [95% CI]	Difference	Difference [95% CI]
Regions				
Capital region of Denmark	—	—	—	—
Central Denmark region	−0.05	0.0 [−0.07; 0.07]	−1.80	−1.01 [−2.93; 0.91]
North Denmark region	−0.03	−0.01 [−0.11; 0.08]	−1.75	−1.45 [−4.1; 1.2]
Region Zealand	−0.02	0.02 [−0.06; 0.1]	0.47	1.58 [−0.66; 3.82]
Region of Southern Denmark	−0.09∗	−0.03 [−0.1; 0.05]	−0.61	1.2 [−0.81; 3.82]
Practice type	∗∗∗	∗∗∗	∗∗∗	∗∗∗
Single-handed	—	—	—	
Cooperative	−0.15∗∗∗	−0.15∗∗∗[−0.22; −0.08]	−3.83∗∗∗	−4.23∗∗∗[−6.29; −2.18]
Partnership	−0.12∗∗∗	−0.12∗∗∗[−0.18; −0.06]	−3.52∗∗∗	−3.59∗∗∗[−5.22; −1.97]
PT-physician ratio^B^				
Low	—	—	—	—
Medium	−0.1	0.01 [−0.06; 0.09]	0.96	1.98 [−0.07; 4.04]
High	−0.04	−0.09 [−0.19; 0.01]	1.99	1.43 [−1.31; 4.18]
PT-employee ratio^B^	∗∗∗	∗∗∗		
Low	—	—	—	—
Medium	−0.02	0.00 [−0.07; 0.08]	−0.47	−0.4 [−2.12; 2.05]
High	0.13	0.14∗∗[0.04; 0.24]	2.96∗	1.87 [−0.90; 4.64]

*P < 0.05,  **P < 0.01,  ***P < 0.001.

^A^A fully adjusted model including all variables listed in the table.

^B^The study population is split into three intervals: 0–15% = low; 16–85% = medium; and 86–100% = high.
